# Elevated Activity in Left Homologous Music Circuits Is Inhibitory for Music Perception but Mediated by Structure–Function Coupling

**DOI:** 10.1111/cns.70174

**Published:** 2024-12-26

**Authors:** Yucheng Wang, Zhishuai Jin, Sizhu Huyang, Qiaoping Lian, Daxing Wu

**Affiliations:** ^1^ Medical Psychological Center, The Second Xiangya Hospital Central South University Changsha Hunan China; ^2^ Medical Psychological Institute of Central South University Changsha Hunan China; ^3^ National Clinical Research Center on Mental Disorders (Xiangya) Changsha Hunan China; ^4^ National Center for Mental Disorders (Xiangya) Changsha Hunan China

**Keywords:** amusia, contralateral homologous region, music perception, regional activity, structure–function coupling

## Abstract

**Aims:**

Previous studies suggested that structural and functional connectivity of right frontotemporal circuits associate with music perception. Emerging evidences demonstrated that structure–function coupling is important for cognition and may allow for a more sensitive investigation of brain–behavior association, while we know little about the relationship between structure–function coupling and music perception.

**Methods:**

We collected multimodal neuroimaging data from 106 participants and measured their music perception by Montreal Battery of Evaluation of Amusia (MBEA). Then we computed structure–function coupling, amplitude of low‐frequency fluctuation (ALFF), gray matter volume (GMV), and structural/functional degree centrality (DC) and utilized support vector regression algorithm to build their relationship with MBEA score.

**Results:**

We found structure–function coupling, rather than GMV, ALFF, or DC, contributed to predict MBEA score. Left middle frontal gyrus (L.MFG), bilateral inferior temporal gyrus, and right insula were the most predictive ROIs for MBEA score. Mediation analysis revealed structure–function coupling of L.MFG, a region that is homologous to typical music circuits, fully mediated the negative link between ALFF of L.MFG and MBEA score.

**Conclusion:**

Structure–function coupling is more effective when explaining variation in music perception. Our findings provide further understanding for the neural basis of music and have implications for cognitive causes of amusia.

## Graphical abstract

We found structure–function coupling, rather than gray matter volume, spontaneous regional neural activity (ALFF) or uni‐modal regional connectivity profile (degree centrality) could predict music perception. Further, structure–function coupling of left middle frontal gyrus (L.MFG), a region that is homologous to typical music circuits, fully mediated the negative link between ALFF of L.MFG and MBEA score.

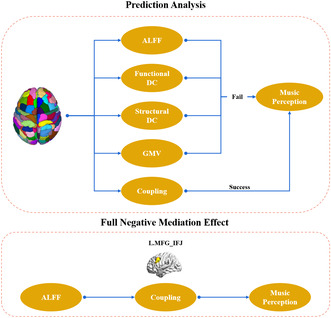

## 1 Introduction

Music is a fundamental but important element in human life. It serves as a means of social communication and contributes to mental health during stressful periods [1]. However, there is a specific impairment called amusia, which is characterized by music perception and/or production deficits but could not be explained by general cognitive or motor deficits. Published articles focused on amusia suggest that structural and functional connectivity of right inferior frontal gyrus (IFG) and superior temporal gyrus (STG), especially connectivity between both regions, engages in music perception [2, 3, 4, 5]. Although structural and functional connectivity separately support efficient neural processing, structure and function are intertwined and coordinated association between them is also important for typical cognition [6, 7, 8, 9]. Currently existing literature only explored the neural basis of music perception via multiple unimodal connectivity features, further exploring the relationship between structure–function association and music perception may help us better understand how the joint variations of structural and functional connectivity contribute to music perception.

Structure–function coupling, a metric that quantifies the association between structure and function, describes how the white‐matter connectivity supports the functional communication between a particular brain region and its interconnected regions. Numerous studies reported the association between multiple clinical outcomes and aberrant structure–function coupling [10, 11, 12, 13]. It is argued that connectivity patterns of specific brain areas are central to understanding their function. Structure–function coupling combines both principles of functional organization (segregation and integration), meanwhile incorporating separate structural and functional connectivity information [14, 15, 16]. This property may allow structure–function coupling to make a more sensitive and exhaustive detection of brain‐cognition association. Indeed, some findings partially support the opinion, they found structure–function coupling contributed to capture subtle association between brain and behavior [17]; also, combination of structural and functional connectivity explained unique variation of cognition [18]. Therefore, compared with other unimodal neural profiles, we argued that structure–function coupling is a more effective feature when capturing neural correlates of music perception.

Machine learning‐based regression algorithm is a novel framework to infer the brain–behavior association. Effectiveness of the method has been validated not only when predicting a broad range of cognitive and behavioral domains but also when using multiple types of neural features (connectivity‐based such as original connectivity value, structural degree centrality [sDC] and functional degree centrality [fDC]; regional neural feature‐based such as amplitude of low frequency fluctuation [ALFF] and gray matter morphology; also including structure–function coupling) to predict [19, 20, 21, 22, 23]. Additionally, differences in performance of prediction models could be used to explain the effectiveness of predictive features for cognition [19, 24, 25]. Thus, such type of regression method is helpful to examine our claim.

In the current study, we aim to investigate whether structure–function coupling associate with music perception and whether structure–function coupling could better capture neural correlates of music perception than other neural features. We hypothesized that (1) interindividual variability in structure–function coupling could explain music perception difference among adults; (2) structure–function coupling is more effective than other neural features when explaining music perception. Comparing with other neural features that are effective in predicting cognitive performance and similar but distinct to structure–function coupling helps to examine the second hypothesis. Given that structure–function coupling derives from and describes connectivity profile of a specific brain region and altered music perception is associated with these unimodal connectivity profiles [26], we chose sDC and fDC (reflect intensity of structural and functional connectivity of a specific area with its interconnected areas, respectively, both also combines segregation and integration principles) as comparable features. Because regional neural activity/morphology is closely related to connectivity [27, 28, 29] and affects music perception [2, 30], we also chose ALFF and gray matter volume (GMV) as comparable features (only reflect segregation principle). We considered these four features as “other neural features” to build prediction model for music perception for each, and compared their predictive performance with model based on structure–function coupling. Additionally, we would conduct exploratory mediation analysis to examine the relationship among music perception and structure–function coupling, sDC, fDC, ALFF, and GMV, which might help to explain the reason why structure–function coupling is more effective than other features when explaining variation in music perception among subjects.

## 2 Methods

### 2.1 Participants

All participants were recruited from Chinese universities in Hunan via campus posters from 2018 to 2022. Inclusion criteria are (1) right‐handed and (2) music training‐naïve. Exclusion criteria include (1) abnormal hearing measured by pure tone audiometry; (2) history of neuropsychiatric disorders; (3) Wechsler Adult Intelligence Scale (WAIS; IQ) score lower than 85; and (4) contraindications for magnetic resonance imaging (MRI). The Ethics Committee of the Second Xiangya Hospital, Central South University approved the research, all participants signed informed consent.

### 2.2 Music Perception Measures

The Montreal Battery of Evaluation of Amusia (MBEA) is a validated tool to measure music perception ability and diagnose congenital amusia [31]. MBEA includes six subtests (scale, contour, interval, rhythm, meter, and memory), and each subtest consists of 30 valid items. Participants need to determine if the paired music is same or different, the meter of music pieces, or if a specific music piece appeared or not in first five subtests. Global averaged score of all subtests was calculated and used as quantitative evaluation of music perception. In the current study, 43 subjects with impaired music perception (MBEA score < 21.5 according to Nan, Sun, Peretz [32]; i.e., congenital amusia) and 64 age‐, sex‐, IQ‐, and education years‐matched subjects with typical music perception were enrolled.

### 2.3 MRI Data Acquisition

All MRI data were collected using a 3 T Siemens Skyra scanner at the Second Xiangya Hospital, Central South University. Resting‐state functional MRI images and T1‐weighted structural images were obtained by echo‐planar imaging and magnetization‐prepared rapid gradient echo sequence. Diffusion MRI was acquired along 64 directions (*b* = 1000s/mm^2^) together with non‐diffusion weighting (*b* = 0 s/mm^2^). See supplementary for detailed scanning parameters.

### 2.4 MRI Data Preprocessing

DPABI 7.0 [33] and FSL 6.0.6 were used to preprocess functional and diffusion MRI data separately. For functional data, main preprocessing steps included: removing first 10 time points, slice timing correction, realign, spatial normalization (resampled to 3 × 3 × 3 mm), smoothing (6 mm smoothing kernel), nuisance regression (linear trend, Friston‐24 head motion parameters, cerebrospinal fluid and whiter matter signals), and band pass filter (0.01–0.1 Hz). For diffusion data, main processing steps included eddy‐current and head motion correction, gradient direction correction, skull stripping (via “BET” command), and extract brain mask from b0 image. T1 images were processed by DPABI with the “New Segment + DARTEL” protocol. One participant with typical music perception was removed due to mean frame‐wise displacement (FD) > 0.2, 106 participants were included in subsequent analysis.

### 2.5 Functional and Structural Connectome

For each subject, functional and structural connectivity matrices were defined using the Brainnetome Atlas [34] which parcellated cerebral cortex into 246 regions of interests (ROI). Pearson correlation of time series between each pair of ROIs was calculated and generated 246 × 246 symmetric matrices. Fisher's *Z* transformation was applied to the matrix representing functional connectivity. For structural connectivity, we first transformed atlas in standard space to native diffusion space according to registration relationship (native diffusion space ↔ native T1 space ↔ standard space). Then, *BedpostX* and *probtrackX2* commands in FSL with default parameters (5000 samples were initiated in each seed voxel) were used to conduct probabilistic tractography. Original asymmetric matrices output following probabilistic tractography were transformed to symmetric form with following formula: (S_ij_ + S_ji_)/5000 × (N_i_ + N_j_), S_ij_ is original streamline counts that streamlines seeded in ROI_i_ and reached ROI_j_, N_i_ is volume of ROI_i_. Further, a consistency‐based method (coefficient of variation of each edge) was applied [35] to reduce spurious connections induced by probabilistic tractography because the criteria contribute to retain consistent both short‐ and long‐range connections across subjects. According to previous studies [35, 36], a 30th percentile was used to remove inconsistent (spurious) structural connections (i.e., inconsistent connections were set to zero).

### 2.6 Structure–Function Coupling, ALFF, sDC, fDC, and GMV

We calculated Spearman correlation coefficient between nonzero structural connectivity and corresponding functional connectivity for each ROI within each subject, which represent structure–function coupling. ALFF, a measure of regional activity intensity, was computed at voxel level within range 0.01–0.1 Hz and then z‐score transformation was applied to origin ALFF and averaged in all 246 ROIs for further analysis. The sum of nonzero structural connectivity and corresponding functional connectivity for each ROI represented sDC and fDC, respectively. GMV of each ROI was extracted from processed gray matter maps.

### 2.7 Estimation of Relationship Between Music Perception and Neuroimaging Features

**FIGURE 1:**
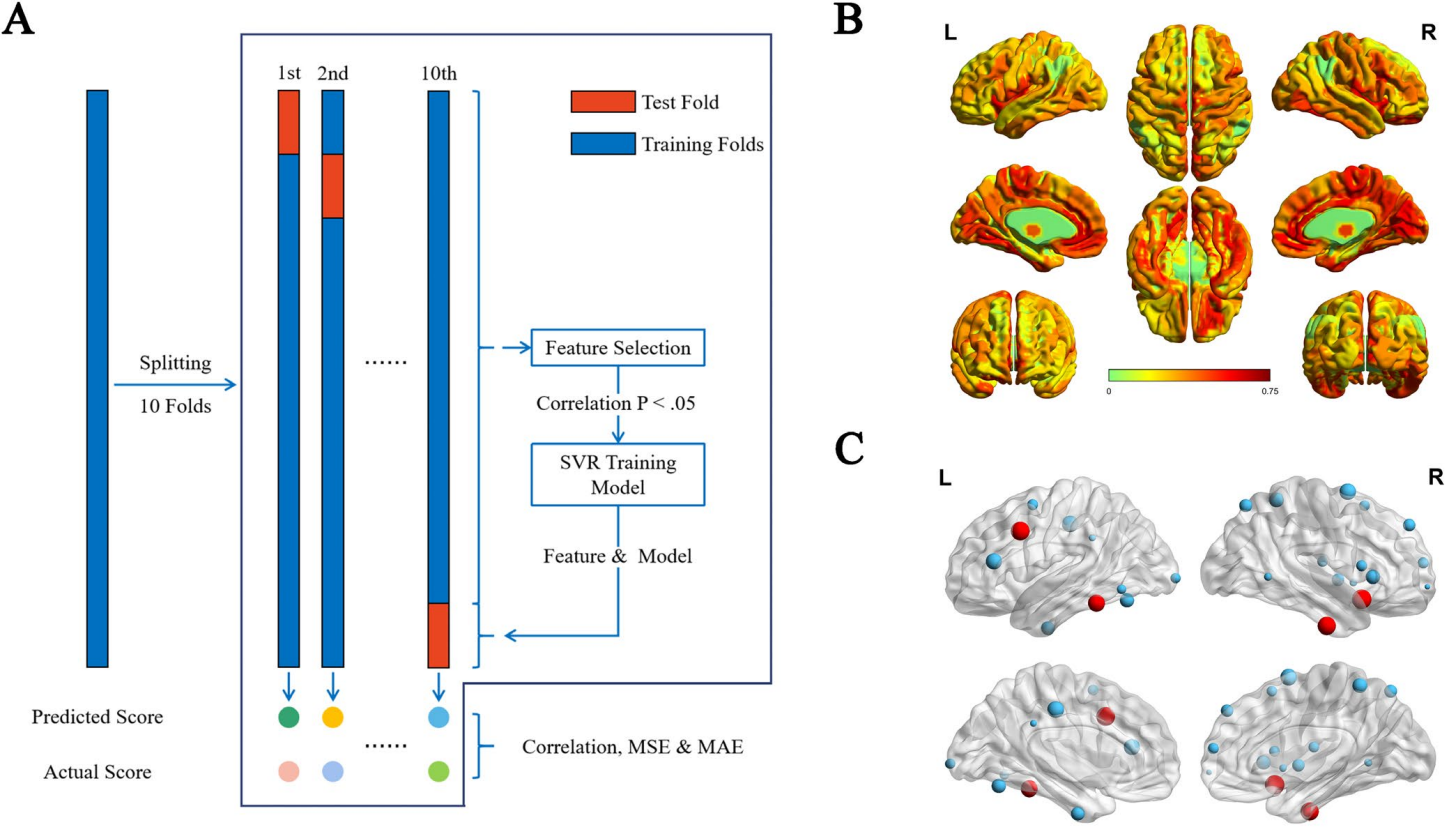
Prediction procedure, spatial pattern of mean structure–function coupling and all predictive ROIs. (A) Illustration of 10‐fold cross validation in our study. In training folds, we filtered potential features (ALFF, functional or structural degree centrality or structure–function coupling) by Spearman correlation and trained prediction models. In each test fold, we calculated predicted MBEA score using features and model from training folds. MAE, MSE and Spearman correlation were used to evaluate prediction performance. (B) Mean structure–function coupling of all participants across cortex. Primary and medial cortex showed relatively high structure–function coupling, while higher‐order and lateral cortex showed relatively low structure–function coupling. (C) All predictive ROIs for MBEA score distribute from subcortical to cortical areas. Greater volume represents greater weights in prediction for each ROI. Most predictive ROIs are marked with red color. L, left; MAE, mean absolute error; MBEA, montreal battery of evaluation of amusia; MSE, mean square error; R, right; ROI, region of interest; SVR, support vector regression.

A machine learning algorithm called L2‐regularized L2‐loss support vector regression (SVR) and a 10‐fold cross validation method were employed to explore the relationship between averaged MBEA score and ALFF, sDC, fDC, GMV, and structure–function coupling, respectively (Figure 1A). We filtered features (Spearman correlation between MBEA score and neural features, uncorrected *p* < 0.05) and generated SVR model with default parameters using random 9 folds of all subjects, then predicted MBEA score in remaining 1‐fold using features and model proposed by training folds. The correlation coefficient, mean absolute error (MAE), and mean square error (MSE) between actual and predicted MBEA score were calculated to evaluate prediction performance. A permutation test (5000 permutations) was performed by randomly reshuffling the original averaged MBEA score to examine the significance of model. The weight of each ROI of each feature was calculated by summing up their weights in each training folds (if a feature was not selected in one training folds, its weight was set to zero in this folds). Only those ROIs that were selected in all cross‐validation folds (i.e., 10 times) and in any predictive model were defined as most predictive ROIs for MBEA score and were included in further analysis and discussion.

### 2.8 Exploratory Analysis

Mediation analysis was employed to explore the potential relationship among multiple neuroimaging features (independent or mediating variables) and music perception (MBEA score, dependent variable). According to our main results, we considered structure–function coupling as mediating variable, MBEA score as dependent variable and other neural features as independent variables. We conducted mediation analysis using the PROCESS plug‐in in SPSS. We conducted correlation analysis between ROIs features and some irrelevant variables (hearing threshold and IQ) in order to examine the specificity of our results.

### 2.9 Association Between Meta‐Analytic Cognitive Functions and Predictive ROIs

We utilized the *Decoder* function in Neurosynth (https://neurosynth.org/decode/), which allows to quantitatively compare any brain volume to large‐scale synthesis of functional MRI data [37], to describe the potential cognitive functions and implications of the most predictive ROIs for MBEA score. The spatial correlation between these ROIs with or without their connected regions (i.e., map type of statistic is ROI/mask) and meta‐analytic brain map of topical terms in database were calculated.

Our main result considered sex, age, and mean Jenkinson's FD as covariates. We also conducted the procedure above without covariates as a validation analysis.

## 3 Results

### 3.1 Participant Characteristics

All 106 participants (50 males) aged at 18–24 years and their averaged MBEA score ranged from 14.5 to 29.5 (in which controls' score ranged from 21.67 and maximum MBEA score of congenital amusia was 21.5). We did not find any correlation between MBEA score and age, education years, IQ, and head motion (all *p* > 0.05). There was no significant difference in MBEA score between males and females (*p* > 0.05). More detailed characteristics of participants are presented Supplementary (Table S1).

### 3.2 Prediction Performance of Different Neural Features for MBEA Score

**FIGURE 2:**
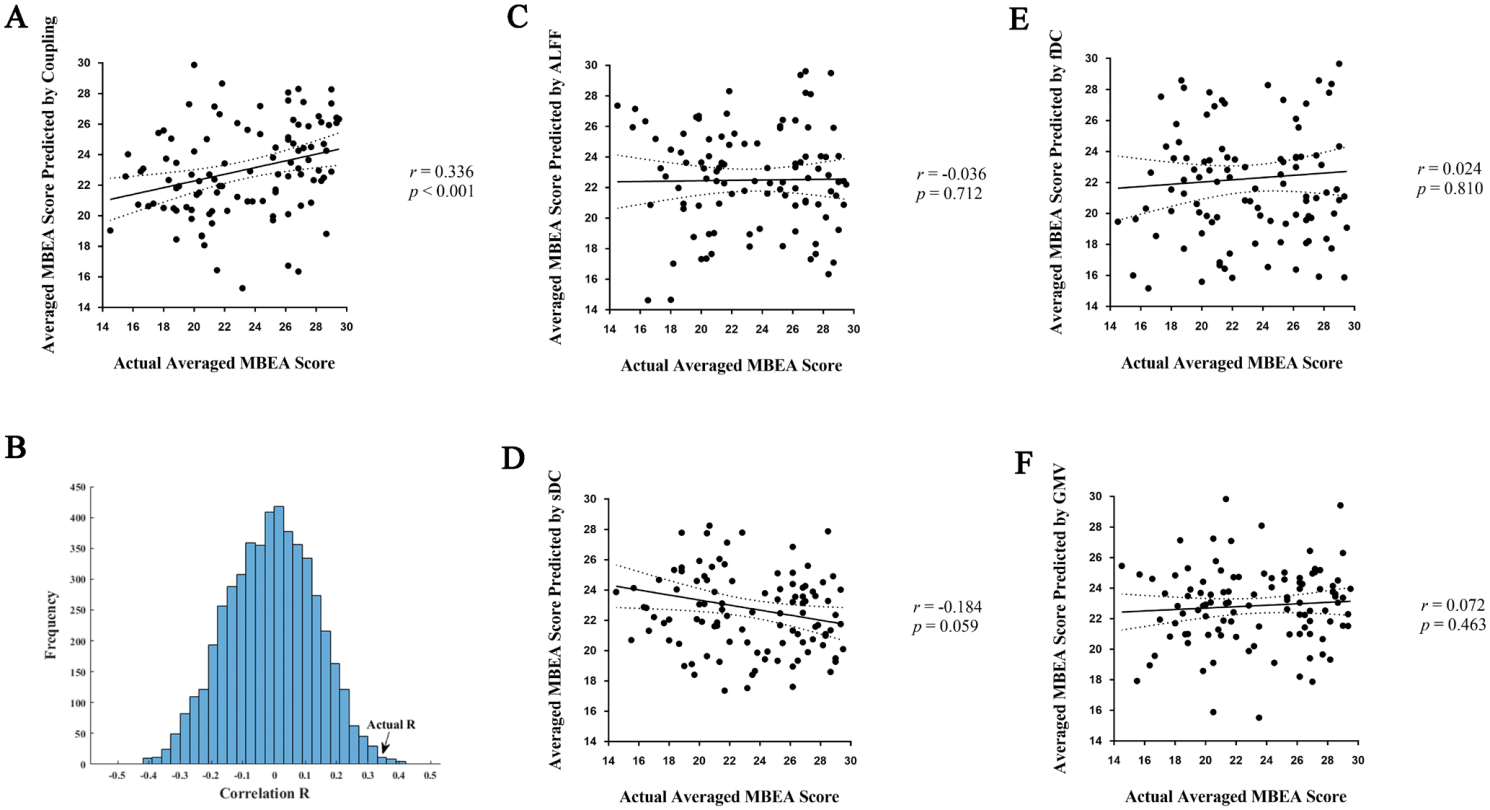
Performance of different feature‐based prediction models. (A) Significant correlation between MBEA score predicted by structure–function coupling based model and actual MBEA score was found (correlation *r* = 0.336, *p* < 0.001). (B) Prediction performance of coupling based model was not spurious according to permutation test (*p* < 0.005). (C–F) Correlation between MBEA score predicted by ALFF, structural, functional degree centrality and GMV and actual MBEA score, respectively. All these four features failed to predict MBEA score (all *p* > 0.05). Solid line and dashed lines in scatter plots represent best‐fit line and 95% confidence interval. ALFF, amplitude of low frequency fluctuation; fDC, functional degree centrality; GMV, gray matter volume; MBEA, montreal battery of evaluation of amusia; sDC, structural degree centrality.

L2‐regularized L2‐loss support vector regression (SVR) and a 10‐fold CV method were employed to explore the relationship between averaged MBEA score and ALFF, sDC, fDC, GMV, and structure–function coupling, respectively (Figure 1A). A set of structure–function coupling (mean structure–function coupling pattern of all participants across cortex was presented in Figure 1B) features showed predictive effect for averaged MBEA score (correlation *r* = 0.336, *p* < 0.001, MAE = 3.487, MSE = 18.263; permutation test *p* = 0.005; Figure 2A,B). Generally, extensive areas including frontal, temporal, parietal, occipital, insula, and basal ganglia in both hemispheres contributed to predict MBEA score (Table 1 and Figure 1C). In which, right intermediate lateral and left caudolateral area 20 of ITG (R.ITG_A20il, L.ITG_A20cl), left inferior frontal junction of MFG (L.MFG_IFJ) and right ventral agranular insula (R.Ins_vIa) were the most predictive ROIs for prediction of MBEA score. SVR model based on other neural features failed to predict MBEA score (*r* = −0.036, −0.184, 0.024, 0.072 for ALFF, sDC, fDC, and GMV, respectively; all correlation *p* > 0.05; Figure 2C–F).

**TABLE 1:** All predictive ROIs and their total weight, selected times and correlation coefficient across all 10 cross validations.

ROI	Selected times in CV	Total weight	Mean correlation *R* (M ± SD)
R.Ins_vIa	10	105.940	0.259 ± 0.034
L.MFG_IFJ	10	89.769	0.301 ± 0.042
R.ITG_A20il	10	70.223	0.249 ± 0.031
L.ITG_A20cl	10	47.495	0.251 ± 0.028
L.CG_A23c	9	42.056	0.229 ± 0.018
R.SFG_A6dl	8	38.669	0.226 ± 0.038
R.SFG_A9l	7	9.756	−0.221 ± 0.033
L.IFG_IFS	6	35.584	0.200 ± 0.033
R.Tha_cTtha	5	4.773	−0.192 ± 0.033
L.FuG_A37mv	4	19.074	0.195 ± 0.024
R.SPL_A7c	4	11.300	0.182 ± 0.032
L.MFG_A6vl	4	5.475	0.191 ± 0.033
R.PCun_A5m	3	21.857	0.185 ± 0.036
R.IFG_A44op	3	19.021	0.150 ± 0.046
L.PhG_A35/36r	2	15.834	0.146 ± 0.047
R.BG_dlPu	2	9.983	0.182 ± 0.027
R.SFG_A10m	2	5.885	−0.185 ± 0.033
L.LOcC_OPC	2	4.428	−0.158 ± 0.032
R.IFG_A44v	1	5.755	0.135 ± 0.038
R.SFG_A8m	1	4.627	0.129 ± 0.041
L.ITG_A37vl	1	3.281	0.119 ± 0.038
R.MTG_A37dl	1	1.533	0.139 ± 0.032
R.BG_vmPu	1	1.065	0.153 ± 0.025
L.CG_A23d	1	0.791	0.137 ± 0.033
R.MFG_A10l	1	0.117	−0.164 ± 0.043

Abbreviations: A10l, lateral area10; A10m, medial area 10; A20cl, caudolateral area 20; A20il, intermediate lateral of area 20; A23c, caudal area 23; A23d, dorsal area 23; A35/36r, rostral area 35/36; A37dl, dorsolateral area 37; A37mv, medioventral area 37; A37vl, ventrolateral area 37; A44op, opercular area 44; A44v, ventral area 44; A5m, medial area 5; A6dl, dorsolateral area 6; A6vl, ventrolateral area 6; A7c, caudal area 7; A8m, medial area 8; A9l, lateral area 9; BG, basal ganglia; CG, cingulate gyrus; cTtha, caudal temporal thalamus; CV, cross validation; dlPu, dorsolateral putamen; FuG, fusiform gyrus; IFG, inferior frontal gyrus; IFJ, inferior frontal junction; IFS, inferior frontal sulcus; Ins, insula; ITG, inferior temporal gyrus; L, left; LOcC, lateral occipital cortex; M ± SD, mean ± standard deviation; MFG, middle frontal gyrus; MTG, middle temporal gyrus; OPC, occipital polar cortex; PCun, precuneus; PhG, parahippocampus gyrus; R, right; ROI, region of interest; SFG, superior frontal gyrus; SPL, superior parietal lobule; Tha, thalamus; vIa, ventral agranular insula; vmPu, ventromedial putamen.

**FIGURE 3:**
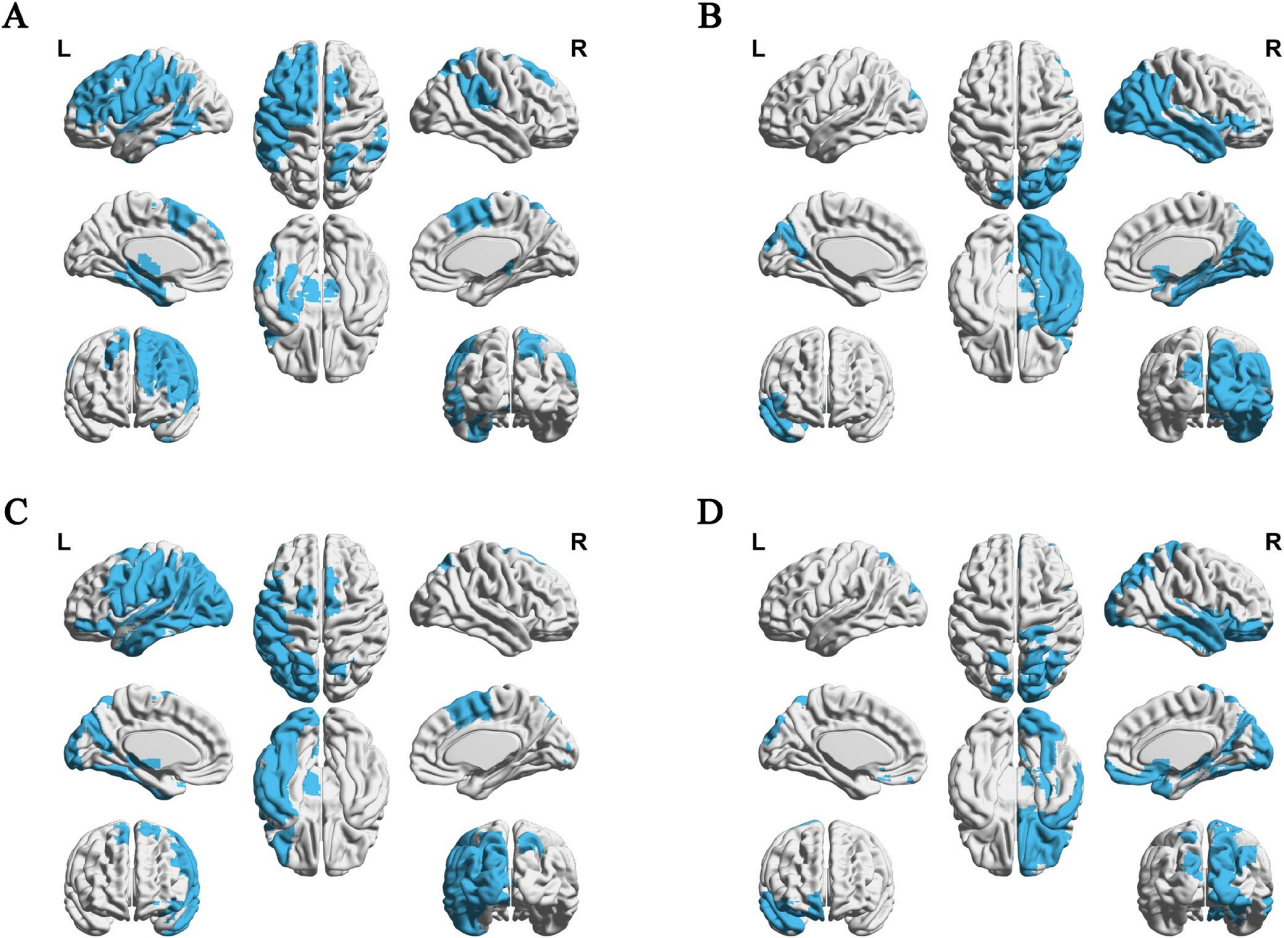
Structural connectivity patterns of the four most predictive ROIs. (A–D) demonstrate connectivity patterns of L.MFG_IFJ, R.ITG_A20il, L.ITG_A20cl and R.Ins_vIa, respectively. All four ROIs mainly connected with their ipsilateral frontotemporal areas and also connected with occipital and parietal lobule. Particularly, L.MFG_IFJ showed a direct structural connectivity with contralateral IPL. A20cl, caudolateral area 20; A20il, intermediate lateral of area 20; IFJ, inferior frontal junction; Ins, insula; IPL, inferior parietal lobule; ITG, inferior temporal gyrus; L, left; MFG, middle frontal gyrus; R, right; ROI, region of interest; vIa, ventral agranular insula.

All four most predictive ROIs mainly connected with frontotemporal areas of their ipsilateral hemisphere and also connected with other regions such as occipital, parietal, insula lobe, and subcortical regions. Although in the contralateral hemisphere, L.MFG_IFJ showed a direct structural connectivity with right inferior parietal lobe (IPL), which is a major node of dorsal music circuits. Structural connectivity patterns of these four regions are shown in Figure 3.

### 3.3 Mediated Effects and Specificity of Structure–Function Coupling

**FIGURE 4:**
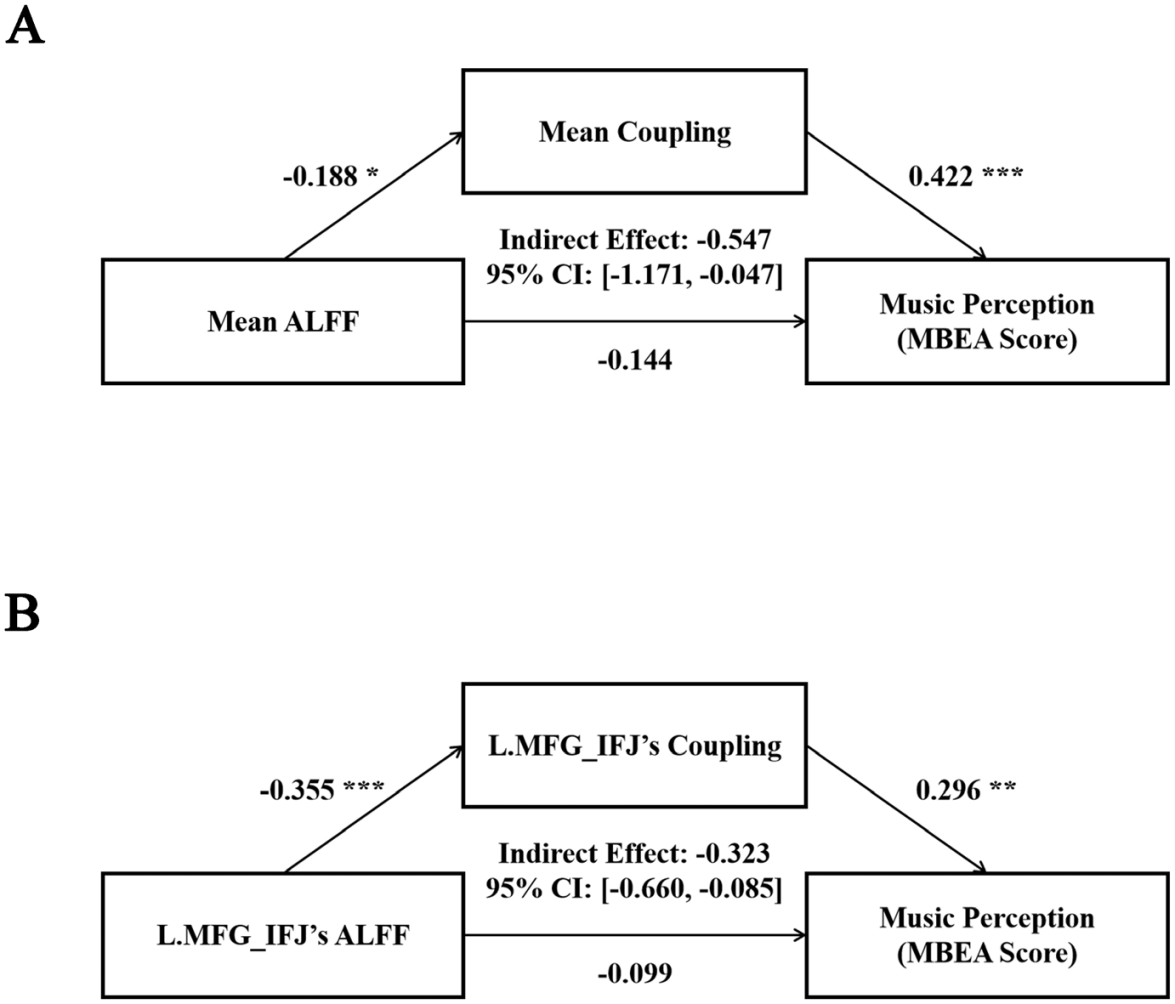
Mediated relation among ALFF, structure–function coupling and MBEA score. (A) Mean structure–function coupling of all four most predictive ROIs fully mediated the relation between mean ALFF and MBEA score (partially standardized indirect effect = −0.547). (B) Structure–function coupling of L.MFG_IFJ fully mediated the relation between ALFF and MBEA score (partially standardized indirect effect = −0.323). ALFF, amplitude of low frequency fluctuation; CI, confidence cnterval; IFJ, inferior frontal junction; L, left; MBEA, montreal battery of evaluation of amusia; MFG, middle frontal gyrus; ROI, region of interest. *p < 0.05; **p < 0.01; ***p < 0.001.

Following the predictive effects of structure–function coupling on MBEA score reported above, we employed exploratory mediation analysis to explain the multiple relationship among neural features and MBEA score. We found a full indirect effect of mean ALFF on music perception through mean structure–function coupling (partially standardized indirect effect = −0.547, 95% CI: [−1.171, −0.047]; Figure 4A) of the most stable four ROIs. Separately, we captured this type of effect on L.MFG_IFJ (partially standardized indirect effect = −0.323, 95% CI: [−0.660, −0.085]; Figure 4B).

We did not find any relation of structure–function coupling of the most effective four ROIs to IQ and hearing threshold (all *p* > 0.05).

### 3.4 Meta‐Analytic Cognitive Functions Related to Predictive ROIs

**FIGURE 5:**
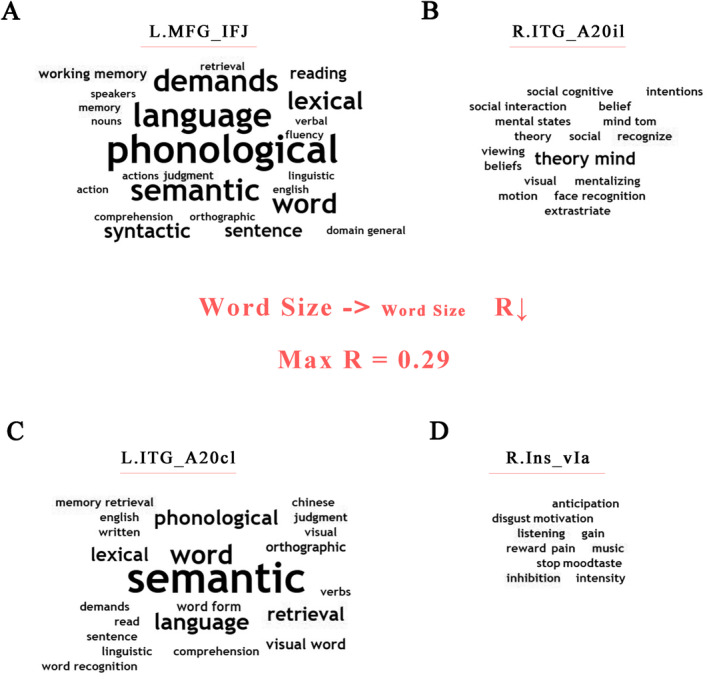
Cognitive meaning of most predictive ROIs for MBEA score with their connected regions. Word clouds (A–D) presented related cognitive functions of L.MFG_IFJ, R.ITG_A20il, L.ITG_A20cl and R.Ins_vIa, respectively. Greater word size represents stronger spatial correlations between regions and cognitive terms in Neurosynth (“phonological” showed max correlation coefficient, 0.29). A20cl, caudolateral area 20; A20il, intermediate lateral of area 20; IFJ, inferior frontal junction; Ins, insula; ITG, inferior temporal gyrus; L, left; MFG, middle frontal gyrus; R, right; ROI, region of interest; vIa, ventral agranular insula.

The four most predictive ROIs were associated with several meta‐analytic cognitive terms such as language, phonological, semantic, memory, retrieval, judgment, cognitive/executive control and music. In Figure 5, we illustrated cognitive terms of the four ROIs with their connected regions, and some interesting terms potentially associated with music perception were marked as red. Greater word size indicated stronger spatial correlations between regions and cognitive terms. Cognitive terms of the four ROIs only were presented in Supporting Information (Tables S3 and S4).

### 3.5 Validation Analysis

Except mediating effect of mean structure–function coupling of most predictive ROIs for MBEA score, similar results were found when we considered age, sex and head motion as covariates or not. Results of validation analysis were shown in Supporting Information (Figures S1—S3, Table S2).

## 4 Discussion

Understanding the neural basis of music processing is an important issue of cognitive neuroscience. In this study, we first investigated the relationship between music perception and structure–function coupling. And we found widely distributed structure–function of brain regions from cortical to subcortical, rather than other neural features, predicted music perception ability. Structure–function coupling of L.MFG_IFJ, L.ITG_A20cl, R.ITG_A20il, and R.Ins_vIa made the most effective contributions for prediction. Mean structure–function coupling of four regions above, especially L.MFG_IFJ, fully mediated the relation between their spontaneous neural activity intensity and music perception.

Right insula is a key node of ventral structural pathway between IFG and STG, previous studies indicated insula engaged in multiple musical cognition [38, 39, 40] and altered structure or function in right insula might disturb the neural activity of right ventral stream of music neural processing. Indeed, right insula is the core of lesion areas causing acquired amusia [3]. We found decreasing structure–function coupling of R.Ins_vIa contributed to worse music perception, which is consistent with opinion above. ITG is another brain region of frontotemporal network and is connected with STG [41]. Aberrant structure or function of ITG also involved in amusia [3]. We also found a positive relation between structure–function coupling of R.ITG_A20il and music perception. These results provide further support for the role of right frontotemporal circuit in music perception [2, 3, 42].

We found structure–function coupling of wide brain regions could predict music perception, but ALFF, GMV, and both DC failed to do so. Structure–function coupling is implicit in structural constraint, maintenance and modulation for function of certain brain regions or circuits [43, 44]. These results indicated that uncoordinated/disorganized neural activity arising from decoupled structure and function, rather than aberrant regional activity, volume or connectivity underlies music perception. High plasticity of human brain and/or interaction between structure and function might bring unpredictable changes either in structure or function across lifespan, which increases the difficulty and lead to failure when exploring brain‐behavior association via single modal neuroimaging features. Structure–function coupling could capture this gap, therefore, structure–function coupling is predictive for music perception in our study and might be more effective when understanding other cognitive functions. Moreover, we found mean structure–function coupling of most predictive ROIs fully mediated the relation between ALFF and music perception, which further explain the reason why regional activity failed to predict music perception: elevated spontaneous regional neural activity leads to poor music perception by disrupting the coordinated relationship between structure and function.

Particularly, we found a fully negative indirect effect of L.MFG_IFJ's ALFF on music perception. Classical view argued that upregulating changes of contralateral hemispheric homologous areas of certain hypofunction regions play a compensatory role [45, 46, 47]. Impaired music perception is associated with abnormal structure and function of right IFG. MFG_IFJ is adjacent to IFG, and decreased activity of right MFG_IFJ contributed to impaired music perception as well [48, 49]. L.MFG_IFJ is structurally connected with and homologous to music circuits, the negative association between activity of L.MFG_IFJ and music perception was inconsistent with the classical view. Some evidences from aphasia also indicated that the up‐regulation in contralateral hemisphere might be maladaptive. Keser et al. indicated that poor language recovery after stroke was associated with higher tract integrity of right AF [50]. More causally, Ren et al. [51] suggested that inhibitory transcranial magnetic stimulation (TMS) targeted in right contributed to recovery of aphasia and demonstrated comparable treatment efficacy to excitatory TMS therapy targeted in left superior frontal gyrus. Neural basis of aphasia/language is left‐dominant and homologous with amusia/music, these findings have certain reference value for our study. Several studies have reported increased inter‐hemispheric functional connectivity in congenital amusia at both task‐ and resting state, and argued that abnormal cooperation between hemispheres is associated with impaired music perception [52, 53, 54, 55]. Our findings further suggested that excessive involvement of the left hemisphere might also disrupt normal cooperation and thus be detrimental to music perception.

We can draw some inferences about cognitive causes at least for congenital amusia according to our results and cognitive neuroscience knowledge. Anterior insula (aIns) is the integral core hub of salience network, which assists target brain regions to generate appropriate cognitive responses and guide behavior to salient stimuli [56]. When salient stimulations are detected, aIns participate in modulating autonomic reactivity and facilitating access to attention and working memory resources. Meanwhile, we found altered structure–function coupling underlay music perception and the most predictive ROIs for music perception involved in cognitive functions such as memory (working memory, memory retrieval), cognitive control and judgment according to Neurosynth. Therefore, we argue that abnormal attention and/or abnormal memory storage and/or retrieval for music stimulations may be potential cognitive causes of amusia, which agree with neurobiology framework of auditory learning [57] and amusia [2].

There are several limitations of present study. First, the small sample size of our study and lack of independent test data set limits the generalizability of our findings. Second, although resting‐state functional connectivity reflects spontaneous function communications unconstrained by specific cognitive components, the estimation of functional connectivity during music processing seems to enhance differences of neural circuitry underlying music perception between subjects [58]. Therefore, estimating functional activity and structure–function coupling from task‐state fMRI helps to further highlight our findings. Thirdly, we found all predictive ROIs for music perception distributed in both hemispheres, and number/weights of ROIs in the left hemisphere are similar to those ROIs in the right hemisphere. Structure–function coupling of bilateral ITG also positively correlated with music perception, these findings disagree with right‐lateralized opinion of musical neural processing. Pitkäniemi et al. [59] suggested singing music lean on left lateralized pathways. Neural lateralization of music needs further investigation. In addition, we gave some “brave” assumptions about cognitive causes of music processing although they had been proven to a certain extent [49, 52, 60]. All these findings and assumptions needs further exploration and validation.

## 5 Conclusions

Our findings indicate that structure–function coupling is more effective to capture neural correlates of music, and increased activity of contralateral regions homologous with music pathways account for worse music perception via decoupling the association between structure and function. Altogether, our findings propose a new perspective to neural basis of music and plasticity, and offer insights to psychological processing of music.

## Author Contributions

Zhishuai Jin, Sizhu Huyang, and Qiaoping Lian participated in the recruitment of subjects, and collected data. Yuchen Wang involved in the data analysis, design, and development of methodology, visualization of results performed, and wrote the initial draft. Daxing Wu designed the study, guided writing initial draft, and revised the main manuscript. All authors reviewed and approved the manuscript.

## Ethics Statement

Our study was approved by Ethics Committee of the Second Xiangya Hospital of Central South University. All participants provided informed consent through their signatures.

## Conflicts of Interest

The authors declare no conflicts of interest.

## Supporting information


Data S1.

## Data Availability

All data that supports these findings of current study are available on request from the corresponding author upon reasonable request.
