# Supplementary material for: Elevated Activity in Left Homologous Music Circuits Is Inhibitory for Music Perception but Mediated by Structure–Function Coupling
Source: CNS Neurosci Ther. 2024 Dec 26;30(12):e70174. doi: 10.1111/cns.70174 (PMC11671239; doi:10.1111/cns.70174)
Supplement: Supplementary file 1 — Data S1. [file CNS-30-e70174-s001.docx]

**Supplementary Methods**

Scanning parameters for T1-weighted structural images: repetition time = 1900 ms, echo time = 2.03 ms, slices = 176, slice thickness = 1 mm, acquisition matrix = 256 × 256, flip angle = 9° and voxel size = 1 × 1 × 1 mm^3^.

Scanning parameters for resting-state functional MRI images: repetition time = 2000 ms, echo time = 30 ms, slices = 32, slice thickness = 4 mm, acquisition matrix = 256 × 256, flip angle = 80° and voxel size = 4 × 4 × 4 mm^3^, measurements = 216.

Diffusion MRI was acquired along 64 directions (b = 1000s/mm^2^) together with non-diffusion weighting (b = 0s/mm^2^) with following parameters: repetition time = 7000 ms, echo time = 86 ms, slices = 60, slice thickness = 2 mm, acquisition matrix = 128 × 128, flip angle = 90° and voxel size = 2 × 2 × 2.5 mm^3^. Duration of resting-state functional, T1 and diffusion MRI sequence is 7min 16s, 8min 8s and 10min 58s respectively.

**Supplementary Results**

**Table S1.** Demographic and Cognitive Characteristics of All Participants.

| Group  Variables | CA (*n* = 43)  (M ± SD) | NA (*n* = 63)  (M ± SD) | P Value |
| --- | --- | --- | --- |
| Age (Years) | 19.580 ± 2.185 | 20.050 ± 1.930 | .250 |
| Sex (Male/Female) | 23/20 | 27/36 | .282 |
| Education (Years) | 13.670 ± 1.286 | 14.14 ± 1.693 | .128 |
| IQ | 105.520 ± 8.548 | 107.845 ± 8.688 | .238 |
| Mean Head Motion (FD Jenkinson) | .060 ± .030 | .069 ± .032 | .152 |
| Averaged MBEA Score | 19.155 ± 1.805 | 26.069 ± 2.243 | < .001 |
| Scale Score | 18.860 ± 2.541 | 26.238 ± 3.115 | < .001 |
| Contour Score | 19.209 ± 3.488 | 27.206 ± 2.294 | < .001 |
| Interval Score | 18.698 ± 3.335 | 25.873 ± 3.617 | < .001 |
| Rhyme Score | 19.302 ± 3.004 | 25.984 ± 3.103 | < .001 |
| Meter Score | 19.047 ± 3.760 | 23.587 ± 4.137 | < .001 |
| Memory Score | 19.814 ± 3.724 | 27.524 ± 2.501 | < .001 |
| Hearing Threshold | 77.050 ± 57.826 | 63.02 ± 63.636 | .272 |

**Abbreviations:** MBEA: Montreal Battery of Evaluation of Amusia; M ± SD: Mean ± Standard Deviation; CA: Congenital Amusia; NA: Non-amusia; FD: Frame-wise Displacement

**
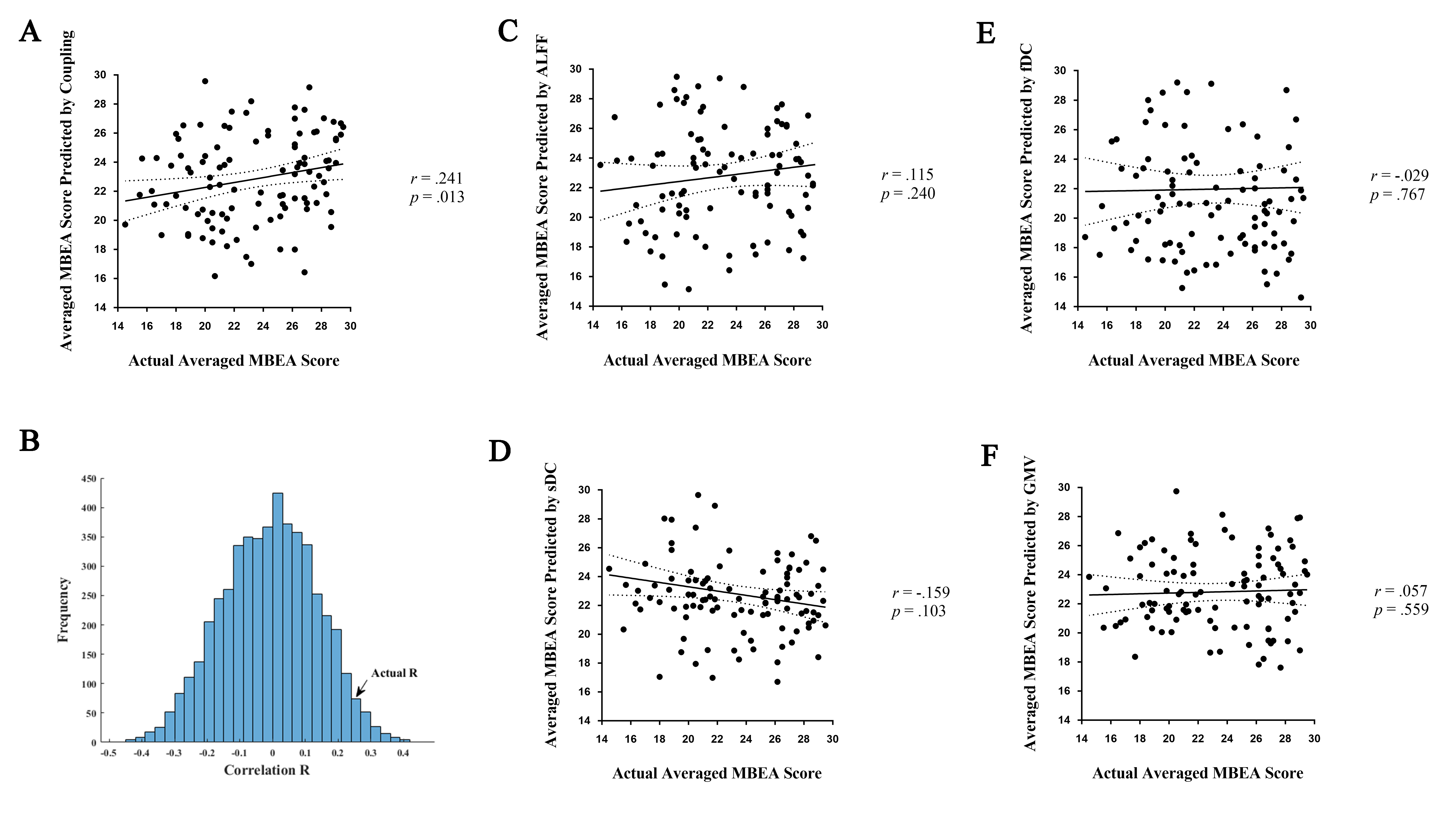
Figure S1. Performance of different feature-based prediction model without covariates.** Same with our main results, **(A)** Actual MBEA score could be predicted by structure-function coupling based model (correlation *r* = .241, *p* = .013; MAE = 3.749, MSE = 19.681). **(B)** Prediction performance of coupling based model was not accident according to permutation test (*p* = .035). **(C), (D), (E) and (F)** Correlation between MBEA score predicted by ALFF, structural, functional degree centrality and GMV and actual MBEA score respectively. All these four features also failed to predict MBEA score (all correlation *p* > .05). Solid line and dashed lines in scatter plots represent best-fit line and 95% confidence interval.

**Abbreviations:** MBEA: Montreal Battery of Evaluation of Amusia; ALFF: Amplitude of Low Frequency Fluctuation; sDC: structural Degree Centrality; fDC: functional Degree Centrality; GMV: Gray Matter Volume.


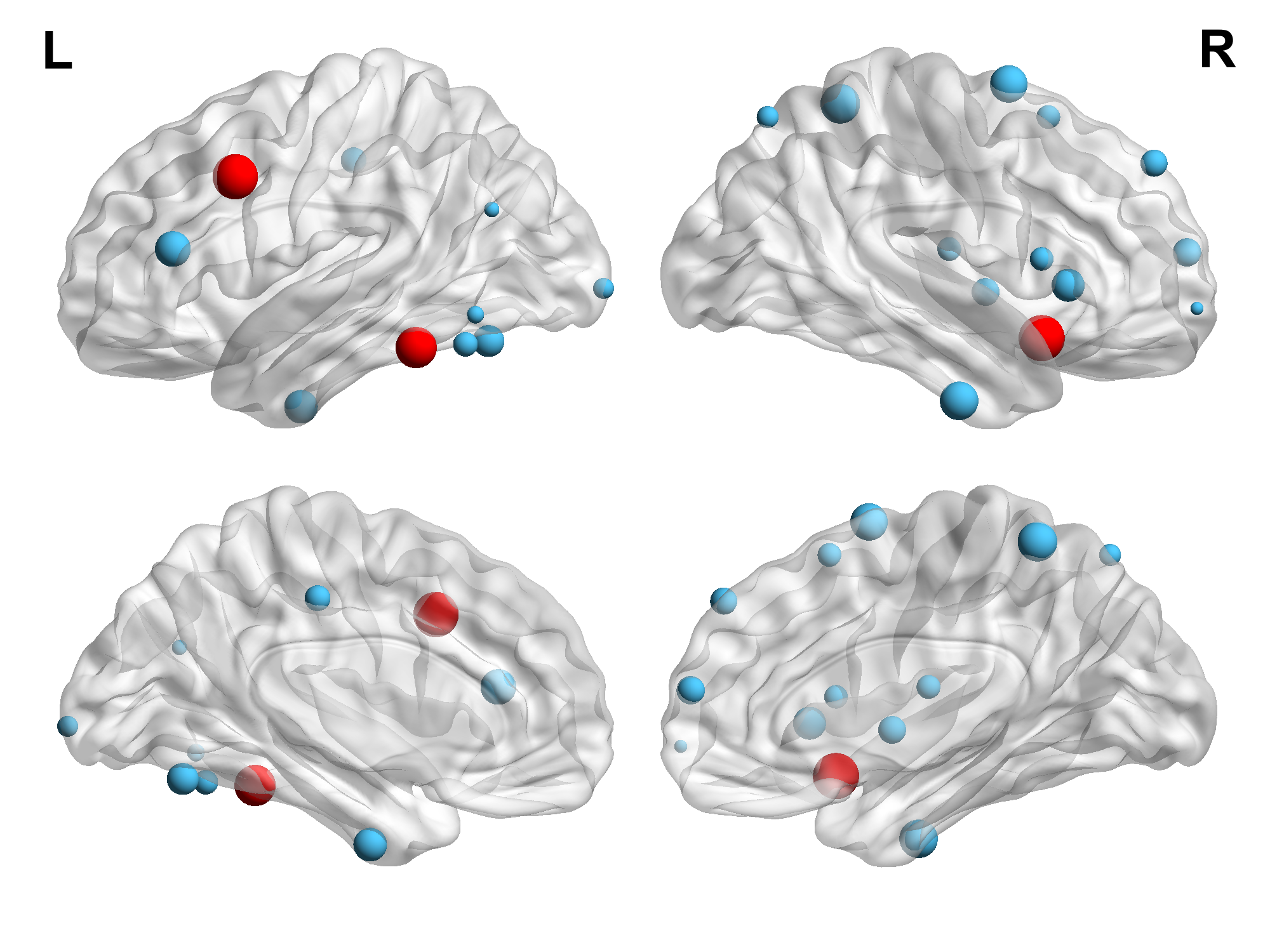


**Figure S2. All predictive ROIs when conducting prediction without covariates.** Greater volume represents greater contribution in prediction for each ROI. Most effective ROIs are marked with red color. Except R.ITG_A20il, most predictive ROIs are same with our main results.

Spearman Correlation: Structure-function Coupling

**Abbreviations:** ITG: Inferior Temporal Gyrus; Ins: Insula; A20il: Intermediate Lateral of Area 20; ROI: Region of Interest; R: Right.


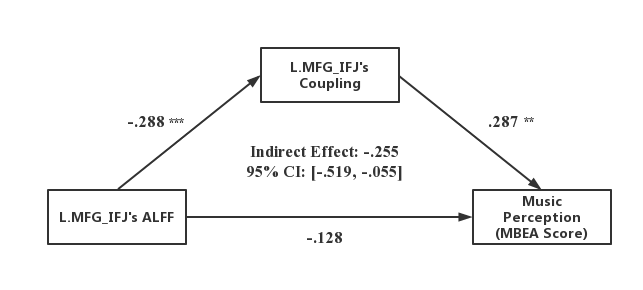
**Figure S3. Mediated relation among ALFF, structure-function coupling and MBEA score.** Structure-function coupling of L.MFG_IFJ fully mediated the relation between ALFF and MBEA score (partially standardized indirect effect = -.255).

**Abbreviations:** MFG: Middle Frontal Gyrus; IFJ: Inferior Frontal Junction; L: Left; MBEA: Montreal Battery of Evaluation of Amusia; ALFF: Amplitude of Low Frequency Fluctuation; CI: Confidence Interval.

**Table S2.** All predictive ROIs and their total weight, selected times and correlation coefficient across all 10 cross validations when covariates were ignored

| **ROI** | **Selected Times in CV** | **Total Weight** | **Mean Correlation R**  **(M ± SD)** |
| --- | --- | --- | --- |
| R.Ins_vIa | 10 | 115.139 | 0.254 ± 0.035 |
| L.MFG_IFJ | 10 | 106.076 | 0.277 ± 0.035 |
| L.ITG_A20cl | 10 | 61.708 | 0.250 ± 0.026 |
| R.SFG_A6dl | 7 | 36.196 | 0.229 ± 0.037 |
| R.ITG_A20il | 6 | 41.641 | 0.214 ± 0.031 |
| R.Tha_cTtha | 6 | 5.276 | -0.207 ± 0.034 |
| R.PCun_A5m | 5 | 48.045 | 0.199 ± 0.032 |
| L.IFG_IFS | 5 | 29.176 | 0.198 ± 0.035 |
| R.IFG_A44op | 3 | 19.671 | 0.172 ± 0.042 |
| L.FuG_A37mv | 3 | 17.411 | 0.194 ± 0.02 |
| R.SFG_A10m | 3 | 10.341 | -0.187 ± 0.034 |
| R.SFG_A9l | 3 | 8.646 | -0.176 ± 0.031 |
| L.PhG_A35/36r | 2 | 21.796 | 0.154 ± 0.044 |
| R.BG_dlPu | 2 | 9.619 | 0.177 ± 0.029 |
| L.CG_A23c | 2 | 7.306 | 0.187 ± 0.017 |
| R.SPL_A7c | 2 | 3.959 | 0.174 ± 0.032 |
| L.LOcC_OPC | 2 | 3.369 | -0.164 ± 0.032 |
| R.MFG_A10l | 2 | 0.569 | -0.166 ± 0.038 |
| L.ITG_A37elv | 1 | 6.132 | 0.115 ± 0.049 |
| R.SFG_A8m | 1 | 5.215 | 0.142 ± 0.042 |
| R.IFG_A44v | 1 | 5.106 | 0.138 ± 0.032 |
| L.ITG_A37vl | 1 | 1.775 | 0.141 ± 0.042 |
| L.IPL_A39rv | 1 | 1.077 | 0.136 ± 0.034 |

**Abbreviations:** ROI: Region of Interest; M ± SD: Mean ± Standard Deviation; CV: Cross Validation; Ins: Insula; MFG: Middle Frontal Gyrus; ITG: Inferior Temporal Gyrus; CG: Cingulate Gyrus; SFG: Superior Frontal Gyrus; IFG: Inferior Frontal Gyrus; Tha: Thalamus; FuG: Fusiform Gyrus; SPL: Superior Parietal Lobule; PCun: Precuneus; PhG: Parahippocampus Gyrus; BG: Basal Ganglia; LOcC: lateral Occipital Cortex; IPL: Inferior Parietal Lobule;

A20il: Intermediate Lateral of Area 20; A20cl: Caudolateral Area 20; vIa: Ventral Agranular Insula; IFJ: Inferior Frontal Junction; A23c: Caudal Area 23; A6dl: Dorsolateral Area 6; A9l: Lateral Area 9; IFS: Inferior Frontal Sulcus; cTtha: Caudal Temporal Thalamus; A37mv: Medioventral Area 37; A7c: Caudal Area 7; A5m: Medial Area 5; A44op: Opercular Area 44; A35/36r: Rostral Area 35/36; dlPu: Dorsolateral Putamen; A10m: Medial Area 10; OPC: Occipital Polar Cortex; A44v: Ventral Area 44; A8m: Medial Area 8; A37vl: Ventrolateral Area 37; A23d: Dorsal Area 23; A10l: Lateral Area10; A39rv: Rostroventral Area 39; A37elv: Extreme Lateroventral Area37; L: Left; R: Right.

**Table S3.** Spatial correlation of R.ITG_A20il/L.MFG_IFJ and cognitive terms in NeuroSynth

| R.ITG_A20il Only | | L.MFG_IFJ Only | |
| --- | --- | --- | --- |
| Cognitive Terms | Spatial Correlation | Cognitive Terms | Spatial Correlation |
| concentration | 0.061 | judgment | 0.197 |
| prospective | 0.057 | demands | 0.161 |
| expertise | 0.056 | cognitive control | 0.152 |
| intentions | 0.055 | language | 0.133 |
| watching | 0.043 | memory | 0.133 |
| theory mind | 0.030 | retrieval | 0.132 |
| social | 0.027 | word | 0.129 |
| light | 0.027 | verbal | 0.125 |
| happy faces | 0.026 | stroop | 0.119 |
| learn | 0.025 | working memory | 0.117 |
| compulsive disorder | 0.024 | lexical | 0.113 |
| experiences | 0.022 | linguistic | 0.111 |
| atrophy | 0.021 | semantic | 0.106 |
| videos | 0.021 | phonological | 0.101 |
| obsessive | 0.020 | rule | 0.099 |
| amnestic | 0.020 | sentence | 0.099 |
| obsessive compulsive | 0.020 | memory retrieval | 0.095 |
| personality | 0.018 | english | 0.093 |
| instructions | 0.018 | instruction | 0.084 |
| mild cognitive | 0.017 | bilinguals | 0.077 |
| major depression | 0.016 | syntactic | 0.066 |
| recall | 0.016 |  |  |
| parkinson disease | 0.015 |  |  |

**Abbreviations:** ITG: Inferior Temporal Gyrus; MFG: Middle Frontal Gyrus; A20il: Intermediate Lateral of Area 20; IFJ: Inferior Frontal Junction; L: Left; R: Right.

**Table S4.** Spatial correlation of R.Ins_vIa/L.ITG_A20cl and cognitive terms in NeuroSynth

| R.Ins_vIa Only | | L.ITG_A20cl Only | |
| --- | --- | --- | --- |
| Cognitive Terms | Spatial Correlation | Cognitive Terms | Spatial Correlation |
| inhibitory control | 0.169 | subsequent memory | 0.162 |
| inhibition | 0.166 | organization | 0.144 |
| response inhibition | 0.106 | thought | 0.085 |
| taste | 0.071 | semantic memory | 0.067 |
| emotional information | 0.070 | word form | 0.057 |
| stop | 0.059 | amnestic | 0.055 |
| error | 0.054 | word | 0.055 |
| stop signal | 0.051 | verb | 0.051 |
| noxious | 0.041 | visual word | 0.049 |
| anxiety | 0.039 | episodic | 0.04 |
| reactivity | 0.037 | recollection | 0.039 |
| reward | 0.037 | memory | 0.036 |
| disgust | 0.036 | retrieval | 0.034 |
| risk taking | 0.036 | judgment | 0.033 |
| emotional | 0.032 | lexical | 0.032 |
| fear | 0.032 | anxiety disorders | 0.03 |
| losses | 0.030 | encoding | 0.023 |
| threatening | 0.030 | executive control | 0.022 |
| heart | 0.029 | orthographic | 0.022 |
| autonomic | 0.029 | risk taking | 0.021 |
| gain | 0.028 |  |  |
| painful | 0.025 |  |  |

**Abbreviations:** ITG: Inferior Temporal Gyrus; Ins: Insula; A20cl: Caudolateral Area 20; vIa: Ventral Agranular Insula; L: Left; R: Right.
